# Nutritional indicators and metabolic alterations in outpatients with anorexia nervosa: a retrospective study

**DOI:** 10.1007/s40519-021-01121-8

**Published:** 2021-02-19

**Authors:** Enza Speranza, Maurizio Marra, Emilia De Filippo, Carmela De Caprio, Rosa Sammarco, Delia Morlino, Franco Contaldo, Fabrizio Pasanisi

**Affiliations:** Department of Clinical Medicine and Surgery, Internal Medicine and Clinical Nutrition Unit, Federico II University Hospital, Via Pansini 5, 80131 Naples, Italy

**Keywords:** Anorexia nervosa, Hepatic alterations, Nutrition alterations, Phase angle, Malnutrition

## Abstract

**Purpose:**

In patients living with Anorexia Nervosa (AN), dehydration and haemoconcentration, may prevent a correct interpretation of laboratory nutritional parameters. Our study aims to evaluate if some indicators of disease severity, as body mass index (BMI), Phase Angle (PhA) and months of amenorrhea may be predictors of metabolic alterations (serum albumin, liver enzymes).

**Methods:**

In 154 outpatients with AN, case history was collected, and anthropometric and laboratory parameters measured. Patients were divided according to the following tertiles (T) of BMI, duration of amenorrhea and PhA: (1) BMI (T1 < 15.6; T2 15.6–16.8; T3 > 16.8 kg/m^2^); (2) Amenorrhea duration (T1 < 7; T2 7–14; T3 > 14 months); (3) PhA value (T1 < 4.64; T2 4.64–5.35; T3: > 5.35°).

ROC curves were used to determine which of these three indicators (BMI, PhA and amenorrhea duration) might better identify patients belonging to Group A or B (less than 3 or more metabolic abnormalities).

**Results:**

The most frequent registered metabolic alterations were for alkaline phosphatase (ALP), alanine aminotransferase, cholesterol and hemoglobin. Aspartate aminotransferase, ALP and gamma glutamyl transferase abnormalities were frequent in the first tertiles of all the three indicators. Albumin was low in the T1 of BMI and PhA. No differences in nutritional alterations emerged according to amenorrhea duration. PhA had the best performance (AUCs: 0.721) in identifying patients with 3 or more abnormalities, with the optimal cut-off value of 4.5°.

**Conclusions:**

Our data confirmed PhA as the more reliable predictor of metabolic alterations, followed by BMI and amenorrhea duration, especially in the first tertile.

**Evidence-based medicine:**

Level 2.

## Introduction

Anorexia nervosa (AN) is a serious eating disorder that, in contrast to other mental health disorders, is often associated with serious clinical complications. Clinical complications account for more than half of the mortality in these patients [1]. and increase as a result of overall weight loss and duration of the illness [2, 3]. This condition of malnutrition progressively involves any organ, and medical complications can be serious or even life-threatening. Severe starvation induces protein and fat catabolism that leads to loss of cellular volume and function, resulting in functional impairment and structural changes in various organs, including the heart, brain, liver, intestines, kidneys, and muscles [4–6].

In the literature, hepatic complications and some nutritional alterations related to AN are increasingly being described. A previous study demonstrated that abnormal serum concentrations of liver enzymes were a frequent finding not only in hospitalized patients living with AN but also in outpatients [7]. AST (aspartate amino- transferase) and ALT (alanine amino- transferase) were inversely related to weight and BMI. Furthermore, discrepancies between changes in these two enzymes as well as decreased alkaline phosphatase (ALP) and increased GGT (gamma glutamyl transferase) were observed. Regarding some metabolic indicators of nutritional status, in addition to undernutrition, dehydration and haemoconcentration conditions might influence some parameters, such as serum albumin levels. Various studies showed that in patients living with AN, serum albumin and other parameter levels were not a reflection of nutritional status, as indicated by the lack of correlation with BMI values [8, 9].

Several studies have shown that BMI and duration of amenorrhoea play a major role in the severity of this disorder. Furthermore, phase angle (PhA), a parameter derived from bioimpedance analysis (BIA), can be considered an index of the integrity of cell membranes and can be used to evaluate the extracellular/intracellular water distribution: a low PhA (< 5°) is a common finding in severe malnutrition [10, 11]. A retrospective study evaluated the incidence of haematologic complications in a group of 318 AN female patients, suggesting that its incidence was strictly related to the malnutrition duration (i.e., duration of amenorrhea) and the severity of malnutrition defined either by low BMI or PhA [9]. Several studies have also demonstrated a close relationship between PhA, a bioimpedance variable, and nutritional status [12–14].

PhA discriminates between different forms of underweight and is an effective marker of qualitative changes in body composition. A low PhA has also been interpreted as a marker of clinically relevant malnutrition characterized by increased extracellular mass and concomitant reduced body cell mass and functional loss [15].

BMI, amenorrhea duration and PhA are criteria related to a common mechanism of chronic malnutrition state.

The aims of our retrospective cohort study are to evaluate if some indicators of disease severity, as body mass index (BMI), Phase Angle (PhA) and duration of amenorrhea may be predictors of metabolic alterations (serum albumin, liver enzymes) in a group of outpatients living with AN.

## Subjects and methods

154 outpatients with AN who attended the clinic for Eating Disorders at the Clinical Nutrition Unit, Federico II University Hospital in Naples, from 1995 to 2005 were recruited. Our study is a retrospective cohort study; case history, anthropometric and laboratory parameters measured was collected. Patients with restricting subtype (AN-R) according to the criteria of the Diagnostic and Statistical Manual of Mental Disorders (DSM V, 2013) were considered for this study [16]*.*

Liver function tests were performed with routine enzymatic (AST, ALT, ALP, GGT, CHE) assays, and nutritional parameters, such as blood cholesterol (CHOL), glucose (GLU), albumin (ALB), hemoglobin (HB) and lymphocytes (LYM), were evaluated. Upon reviewing clinical records, we collected information about the amenorrhea’s months. Bioimpedance analysis (BIA) was performed at 50 kHz using Human Im Plus II (DS Medica-Milan) at room temperature (22–25 °C) after 20 min in the supine position; all subjects were in a fasted state (12 h) and voided prior to measurement. The measured BIA variables were resistance (*R*), reactance (Xc) and phase angle (PhA). Bioimpedance index (BI-index in cm^2^/ohm) was derived as the ratio height^2^/whole-body R. Using BIA, fat-free mass (FFM) and fat mass (FM) can be estimated by means of predictive equations, which include BIA variables and variables such as age, stature and weight, specific for AN female subjects [10].

Total sample was divided according to the following tertiles (T) of BMI, amenorrhea and PhA:

1. BMI (T1 < 15.6; T2 15.6–16.8; T3 > 16.8 kg/m^2^);
2. Amenorrhea duration (T1 < 7; T2 7–14; T3 > 14 months);
3. PhA value (T1 < 4.64; T2 4.64–5.35; T3: > 5.35°).

The study protocol was approved by the Ethical Committee of the Federico II University Hospital (Prot. N 37/17) and carried out according to the Declaration of Helsinki.

## Statistical analysis

Statistical analyses were performed using SPSS version 19.0 (SPSS, Inc., Chicago, IL, USA). The results are expressed as the mean ± standard deviation (SD). One-way analysis of variance was used for comparisons between subgroups, while Tukey’s test was used for pairwise comparisons. The χ^2^ test was used to assess differences in proportions. A *p* < 0.05 was considered statistically significant.

Receiver-operating characteristic (ROC) curves were used to determine the optimal cut-off value for continuous versus dichotomous variables, to determine which of these three indicators (BMI, PhA and amenorrhea duration) might better identify the highest number of metabolic abnormalities.

As a significant cut-off value was observed, the sensitivity and specificity were presented. While the area under the curve was evaluated, a 5% type-1 error level was called a statistically significant predictive value of the test variables.

## Results

The general characteristics and body composition data obtained with BIA of 154 female AN-R patients (BMI range 13.1–18.5 kg/m^2^, amenorrhea duration range 3–72 months, PhA range 1.9–7.7°) are presented in Table 1.

**Table 1:** General characteristics of 154 restrictive anorectic patients

	mean ± SD	min–max
Age (years)	21.1 ± 4.9	16–38
Weight (kg)	41.8 ± 4.6	29.2–53.5
Stature (cm)	161 ± 5.6	145–174
BMI (kg/m^2^)	16.1 ± 1.4	13.1–18.5
Amenorrhea (months)	13.7 ± 12.9	3–72
FFM (kg)	34.8 ± 4.6	25.4–50.7
FM (kg)	7.1 ± 3.4	0.5–15.7
FM (%)	16.6 ± 7.5	1.4–33
Phase angle (°)	4.93 ± 0.74	2.0–7.80

*FFM* fat-free mass, *FM* fat mass

Anthropometric data expressed for the three tertiles are illustrated in Table 2. Significant differences were observed for body weight and BMI (*p* < 0.05).

**Table 2:** Anthropometric measurements (expressed as mean ± SD) according to BMI, amenorrhea duration and PhA in 154 restrictive anorectic patients

Subgroups	Body mass index			Amenorrhea duration			Phase angle		
	T1, *n* = 52	T2, *n* = 49	T3, *n* = 53	T1, *n* = 52	T2, *n* = 53	T3, *n* = 49	T1, *n* = 51	T2, *n* = 50	T3, *n* = 53
Age (years)	21.2 ± 5.0	20.9 ± 5.2	21.1 ± 4.9	20.1 ± 4.8	21.4 ± 4.0	22.3 ± 5.4	21.2 ± 5.1	20.7 ± 4.7	21.4 ± 5.0
Weight (kg)	37.8 ± 3.6*	41.8 ± 2.9	45.8 ± 3.3	42.4 ± 3.6	42.4 ± 5.0	40.7 ± 5.3**	39.9 ± 4.6*	43.0 ± 4.9	42.6 ± 3.9
Height (cm)	161 ± 6.3	161 ± 5.1	161 ± 5.3	161 ± 4.8	160 ± 5.9	161 ± 6.2	161 ± 5.8	162 ± 6.3	160 ± 4.4
BMI (kg/m^2^)	14.6 ± 0.8*	16.2 ± 0.3	17.6 ± 0.4	16.4 ± 1.3	16.3 ± 1.3	15.7 ± 1.5**	15.5 ± 1.4*	16.3 ± 1.2	16.6 ± 1.3

*T1:* BMI < 15.6 kg/m^2^; amenorrhea duration < 7 months; PhA < 4.64°;  *T2:* BMI 15.6–16.8 kg/m^2^; amenorrhea duration 7–14 months; PhA 4.64–5.35°, *T3:* BMI > 16.8 kg/m^2^; amenorrhea duration > 14 months; PhA > 5.35°

*p ≤ 0.05 versus T2 and T3

**p ≤ 0.05 versus T1 and T2

**Fig. 1:**
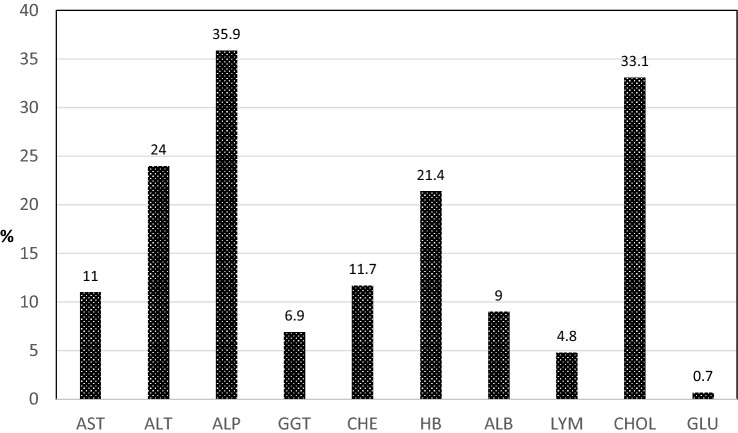
Metabolic abnormalities (%) in 154 restrictive anorectic patients

Distribution of abnormalities’ metabolic in the total sample is presented in Fig. 1. The most frequent ones were expressed for ALP, ALT, CHOL and HB.

### BMI tertiles

A quite high prevalence of nutritional parameters’ abnormalities was observed in T1 patients. T1 had a lower ALB than T2 and T3. A low ALB was significantly higher in T1. GLU was mainly within the normal range, but also in T1, 13.5% of patients showed hypoglycaemia (Table 3).

**Table 3:** Distribution (%) of hemato-biochemical parameters’ abnormalities according to BMI, amenorrhea duration and PhA tertiles in 154 restrictive anorectic patients

Subgroups	Body mass index			Amenorrhea duration			Phase angle		
	T1, *n* = 52	T2, *n* = 49	T3, *n* = 53	T1, *n* = 52	T2, *n* = 53	T3, *n* = 49	T1, *n* = 51	T2, *n* = 50	T3, *n* = 53
Parameter (limits)									
ALB < 3.5 g/dL	21.2*	8.2	11.3	12.7	14.3	14.3	19.6*	12.0	9.4
CHOL > 190 mgdL	42.3	36.7	34.0	38.4	31.0	42.9	37.3	26.0	49.1
HDL < 45 mg/dL	8.6	9.8	10.9	9.6	9.1	10.8	14.8*	6.8	9.8
GLU < 60 mg/dl	13.5*	4.1	7.6	4.8	9.5	10.2	5.9	6.0	11.3
HB < 12.0 g/dL	23.1	20.8	18.9	20	31.0	18.8	29.4***	20.4	13.2
LYM < 1.200 × 10^3^/μL	6.4	2.2	4.2	3.5	5.0	4.7	4.3	4.0	4.3
AST > 35 U/L	9.6	16.3**	5.7	9.5	4.8	16.3*	13.7*	1.0*	7.5
ALT > 35 U/L	23.1	26.5	18.9	28.6***	21.4	15.2	17.6	24.0	26.4***
ALP > 104 U/L	32.6	53.0**	43.4	44.4	38.1	44.9	58.8***	40	30.2
GGT > 36n U/L	13.5***	12.3	1.9	3.2	11.9	14.3***	11.8***	8.0	7.6
CHE < 6.000 U/L	10	16.4	8	14	11.4	9.1	14.6	8.3	12.2

*ALB *albumin, *CHOL *cholesterol, *GLU *fasting blood glucose, *HB *hemoglobin, *LYM *lymphocytes, *ALT *alanine aminotransferase, *AST *aspartate amino transaminase, *ALP *alkaline phosphatase, *GGT *gamma glutamyl transferase, *T1:* BMI < 15.6 kg/m^2^; amenorrhea duration < 7 months; PhA < 4.64°, *T2:* BMI 15.6–16.8 kg/m^2^; amenorrhea duration 7–14 months; PhA 4.64–5.35°, *T3:* BMI > 16.8 kg/m^2^; amenorrhea duration > 14 months; PhA > 5.35°

*p ≤ 0.05 between T1 and T2

**p ≤ 0.05 between T2 and T3

***p ≤ 0.05 between T3 and T1

No significant difference between the BMI subgroups was found for the other parameters (Table 3).

Regarding liver enzymes, Table 3 illustrates statistically significant differences in distribution observed for AST and GGT.

### Amenorrhea duration tertiles

We observed no difference in nutritional alterations, we found a higher hypercholesterolemia distribution (42.9%) 42.9% in T3 (n.s.). Higher values of ALP and GGT was evident in T3 (*p* ≤ 0.005) (Table 3).

### Phase angle tertiles

We observed an increased distribution of nutritional abnormalities in the T1 PhA. Low serum HB was more prevalent in T1 than in T3 (*p* ≤ 0.005); ALB and low HDL cholesterol were significantly higher in T1 than in the other groups (Table 3).

Hepatic alterations were frequent in T1 for AST, ALP and GGT as opposed only to a higher value of ALT in T3 (*p* ≤ 0.005).

### ROC analysis

For this analysis, only six metabolic parameters belonging to the routine haemato-biochemistry evaluation (AST, ALT, ALP, CHE, HB), were selected because they occur in total sample as abnormalities in more than 10%. Patients were divided into two groups based on the number of biochemical abnormalities (Group A ≤ 3; Group B ≥ 3). The hemato-biochemical parameters and body composition data between the groups A and B are presented in Table 4.

**Table 4:** Hemato-biochemical parameters and body composition (expressed as mean ± SD) between the groups A and B

	Group A < 3 abnormalities	Group B ≥ 3 abnormalities
Age (years)	21.2 ± 4.8	21.5 ± 5.9
Weight (kg)	42.1 ± 4.5	41.8 ± 4.9
Stature (cm)	160.6 ± 5.5	162.8 ± 4.3
BMI (kg/m^2^)	16.3 ± 1.3	15.7 ± 1.3
Amenorrhea (months)	13.7 ± 14.0	14.9 ± 13.0
FFM (kg)	37.7 ± 3.6	38.6 ± 4.1
FM (kg)	4.4 ± 1.5	3.2 ± 1.9*
FM %	10.3 ± 2.9	7.4 ± 4.3*
Phase angle (°)	5.0 ± 0.66	4.3 ± 1.0*
AST (U/L)	23.4 ± 9.9	40.3 ± 23.3*
ALT (U/L)	26.7 ± 24.1	67.5 ± 61.1*
ALP (U/L)	86.7 ± 48.9	132.8 ± 103.5*
CHE (U/L)	6469 ± 2067	6806 ± 2303
HB (g/dL)	12.9 ± 1.1	12.2 ± 0.9*
CHOL (mgdL)	179 ± 36	183 ± 42

^*^p ≤ 0.05

**Fig. 2:**
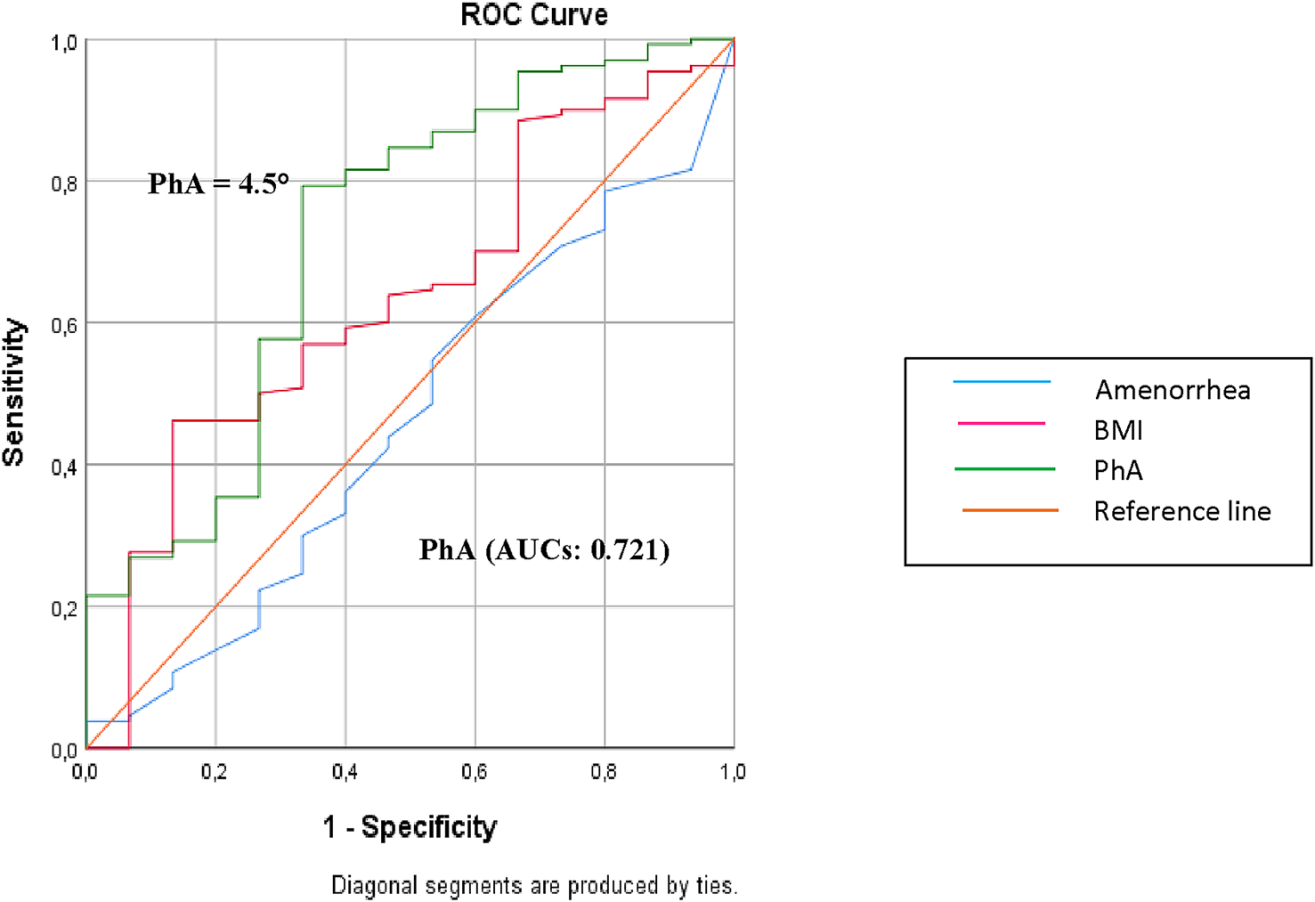
Receiver-operating characteristic (ROC) curve indicator of three or more number of metabolic abnormalities

ROC curves were used to determine which of these three indicators (BMI, PhA and amenorrhea duration) might better identify patients belonging to Group A (less than 3 metabolic abnormalities) or Group B (at least 3 metabolic abnormalities). PhA had the best performance (AUCs: 0.721) in identifying patients with three or more abnormalities, with the optimal cut-off value of 4.5°, whereas BMI and amenorrhea duration proved to be inadequate indicators (AUCs: 0.632 and 0.460, respectively) (Fig. 2).

## Discussion

We evaluated which of these nutritional indicators among BMI, PhA and amenorrhea duration, played a central role in metabolic alterations, especially for some nutritional and liver parameters, in a large cohort of AN-R outpatients.

Our data show that in these patients with chronic malnutrition, albumin is not a good mirror of nutritional status. Analysis of the data indicates that the prevalence of low serum albumin is very similar in different of amenorrhea duration, but a higher prevalence is only related to lower BMI. Our results are in accordance with a systematic review that considered albumin to be an unsuitable nutritional marker in patients with severe caloric restriction, including AN [16, 17].

Although the pathophysiological mechanisms are not completely known, they are considered to be related to the atrophy of fat cells and the gelatinous transformation in bone marrow as a consequence of starvation due to the marked restriction of carbohydrates and fats, which are the source of fat in bone marrow [18]. It remains controversial whether there is a direct correlation between hematological changes and the severity of disease. In a previous study, we reported that these abnormalities might be related to body composition parameters (i.e., PhA and BMI) [9].

As presented by our data, the amenorrhea duration does not have an effect on nutritional alterations, and blood albumin and glycose concentration are the only parameters on which BMI showed an effect. Instead, PhA appears to be a more sensitive issue and seems to play a major role in these alterations unless cholesterolemia is not influenced by a lower PhA.

Our study also showed that AST alterations were changed in all subgroups according to worsening clinical status. PhA had a significant influence on all hepatic enzymes, which were more apparent in the tertiles with the lowest PhA.

ALP changed in the tertiles of both amenorrhea duration and PhA. ALP is often considered to be a hepatic marker, but human serum contains a mixture of ALP isoenzymes from bones, liver and intestines. We can hypothesize that the increased ALP levels observed in AN woman with the worst PhA were probably associated with increased bone turnover due to osteomalacia or secondary osteoporosis, which are frequently observed in these patients [19]. In malnourished patients with AN, marked hypertransaminasemia is recognized as a factor of severity, so it is an indicator of a worse prognosis [4, 7, 20]. These alterations seem to reflect different aspects of impaired liver function. Our study also illustrated that GGT changed in all three subgroups according to the worsening of the illness, but it was not specifically related to the changes in PhA.

In our study, we observed decreased AST and ALT levels, probably linked to the young age of our sample. We also hypothesized that the subjects with more severe disease, as reflected by the lower PhA and a longer duration of illness, would have reached a state of biochemical adaptation. When we verified the AST/ALT ratio, a diagnostic index for alcoholic liver disease, it was greater than 2.0 in only 2% of the total sample, it was less than 1.0 in 50% of the sample, and for the remaining 48%, the ratio ranged from 1.0 to 2.0. This normal ratio confirmed absolutely negligible alcohol intake in these patients.

Even if there have been several studies regarding changes in aminotransferase levels in AN patients, they have shown no consistent patterns in the results. Some studies have revealed abnormal values, but most results lie within normal limits. Other studies have reported that blood liver enzymes increase following refeeding syndrome [21].

According to our previous studies, PhA could be a sensible marker of nutritional status in patients with AN, enabling the extracellular/intracellular water distribution to be identified. Furthermore, recent studies, also conducted by our group, identified PhA as a prognostic predictor of malnutrition risk; those studies demonstrate the potential to use this parameter to monitor treatment because it is a more reliable parameter than BMI for monitoring nutritional state improvement in patients with AN [11, 13]. Our study confirmed the role of PhA as a screening parameter for diagnostic investigations that are not always used in routine clinical practice; a low PhA is as the more reliable predictor of metabolic alterations followed by BMI and amenorrhea duration,

There are some study limitations due to the retrospective design of this study. On the other hand, the major strengths of the study were the well characterized and homogenous study group and body composition data that aptly represent these patients with anorexia nervosa.

### What is already known on this subject?

Dehydration and haemoconcentration in anorexia nervosa may prevent a correct interpretation of laboratory nutritional parameters. In these patients with chronic malnutrition, nutritional and hepatic parameters is not a good mirror of nutritional status, so our study could be identified as some indicators, as body mass index, phase angle and duration of amenorrhea may be predictors of metabolic alterations.

### What does this study add?

Our results enable us to consider the importance of a bioimpedance analysis (BIA) to determine a PhA value to evaluate a state of a chronic malnutrition status in AN-r patients enabling the extracellular/intracellular water distribution to be identified. It confirmed the role of PhA as a screening parameter for diagnostic investigations that are not always used in routine clinical practice; a low PhA is as the more reliable predictor of metabolic alterations followed by BMI and amenorrhea duration.
